# The Role of Learners' Psychological Well-Being and Academic Engagement on Their Grit

**DOI:** 10.3389/fpsyg.2022.848325

**Published:** 2022-02-23

**Authors:** Jiaying Huo

**Affiliations:** College of International Cooperation, Xi'an International University, Xi'an, China

**Keywords:** academic engagement, psychological well-being, positive emotional constructs, pedagogical implications, grit

## Abstract

This review aimed at examining the related studies on the effect of English as a foreign language learners' (EFL) psychological well-being and academic engagement as positive emotional constructs on learners' grit. The positive significant effect of psychological well-being on learners' grit has been confirmed in the literature review. Studies have shown that resilience, enjoyment, engagement, lack of depression, interest, and world meaningfulness can act as mediator variables in the relationship between psychological well-being and grit. Furthermore, few studies have been done on the effect of academic engagement on learners' grit. The studies showed that some factors such as meticulousness, self-control, self-confidence, and motivation act as mediators in the relationship between academic engagement and grittiness. In the end, the pedagogical implications are expounded to promote the quality of language learning quality. This review also provides some suggestions for further research to clarify our perspective over positive emotional variables and their relationships with each other.

## Introduction

Due to the fact that the foreign language learning process was so challenging for some learners, learners' negative emotional constructs such as anxiety, boredom, and burnout (Fathi and Derakhshan, 2019; Fathi et al., 2020a,b; Derakhshan et al., 2021) and their individual differences have been studied in terms of apprehension, personality, motivation, and self-efficacy (Kim et al., 2015). However, the investigation of negative construct effects on learners' academic enjoyment, engagement, and performance was not sufficient. Therefore, positive psychology (PP) has recently emerged and many positive psychologists have verified that the consideration of learners' strength to improve their learning outcome has been effective for many investigators (MacIntyre et al., 2019a; Wang et al., 2021). However, investigators tended to investigate the constructs of positive emotions in more detail with the hope to assist language learners to process language better in their minds (Fang and Tang, 2021). This review is an attempt to investigate the related literature concerned with EFL learners' well-being and academic engagement on learners' grit in classroom contexts. These positive variables have been widely investigated independently. However, the relationships among them can shed light on positive psychology constructs and their correlations with each other.

## Literature Review

### Psychological Well-Being

The objectives of positive psychologists have been changed from subjective happiness to the theory of well-being (Choochom, 2016). Positive psychologists have established numerous theories to elucidate the concept of well-being (Butler and Kern, 2016). Cooke et al. (2016) identified two distinct perspectives in developing well-being construct: hedonic and eudaimonic perspectives. Tuntivivat (2017) indicated that hedonic or subjective well-being highlights subjective knowledge of contentment, life pleasure, and gratification. On the other hand, some components such as having a life purpose, self-realization, positive relationships, spiritual growth, and thriving are the main aspects of eudaimonic or psychological well-being (Seligman, 2011). Eudaimonic underlines both fulfillment and seriousness (Diener et al., 2010). Keyes (2013) argued that psychological well-being promotes following intrinsic objectives, meeting fundamental emotional needs, being aware and doing with consciousness, and performing independently. Earlier investigations have shown that some variables such as socio-demographic variables (Khumalo et al., 2012), hope (Pleeging et al., 2021), self-presentation (Jang et al., 2018), social anxiety (Zongbo et al., 2017), perceived social support (Fan and Lu, 2020), depression, emotional exhaustion, and stress (Jeon et al., 2018), emotional intelligence (Villanueva et al., 2020), and academic resilience (Clough and Strycharczyk, 2012) affect learners' psychological well-being. De Coninck et al. (2019) found out that learners' psychological well-being in high school is significantly related to their levels of psychological well-being in the early days of university. However, they found that psychological well-being declines over time. Furthermore, Burris et al. (2009) found out that learners' optimism is significantly correlated with their high levels of psychological well-being. Howell's (Howell, 2009) study also revealed that psychological well-being can be considered a significant component of learners' academic achievement. Eskisu (2021), in his study, investigated the relationship between learners' parentification, psychological resilience, and psychological well-being. They found a negative correlation between learners' experience of parentification and their psychological well-being and psychological resilience. In studying the role of online positive psychology intervention in non-English learners' psychological well-being, Yurayat and Seechaliao (2021) found the significant effect of online positive psychology intervention on the improvement of learners' psychological well-being.

Although many studies have been done on the role of psychological well-being in education, investigating the role of psychological well-being in EFL instructive environments is still in its initial steps. Moreover, comprehensive studies have been done on EFL instructors' well-being to increase their fulfillment (Huang et al., 2019). However, few studies have been done on EFL learners' psychological well-being. Lan and Saad (2020) found out that learners' psychological well-being and engagement should be emphasized in EFL Chinese context. In another study, Beresnevičiene and Mačianskiene (2000) investigated the effect of learners' psychological well-being and self-esteem on their academic success. Their study revealed that efficacious EFL learners have higher levels of psychological well-being and self-esteem. Moreover, they frequently use memory, cognitive, and social strategies. Moradi and Langroudi (2013) also investigated the role of EFL learners' intrinsic and extrinsic religious orientation, self-esteem, and psychological well-being on their language learning proficiency. They found a significant positive correlation between EFL learners' psychological well-being and their language proficiency. Chen and and Zhang (2020) investigated the role of psychological well-being in EFL learners' overall language performance along with their language skills. They found a significant correlation between EFL learners' psychological well-being, overall language proficiency, and their listening skill.

### Learners' Academic Engagement

According to Lamborn et al. (1992), academic engagement is described as “learners' psychological effort and investment toward learning, understanding, or mastering the skills, crafts, or knowledge that the coursework is intended to promote” (p. 13). However, Skinner et al. (2009) defined academic engagement as “the quality and quantity of students' participation or connection with the educational endeavor and hence with activities, values, individuals, aims, and place that comprise it” (p. 495). Hiver et al. (2021) also argued that the learner engagement construct is multifaceted and includes numerous features such as emotional, cognitive, and behavioral aspects. They maintained that these features interact with each other to determine learners' optimism toward the learning process. Dincer et al. (2019) stated that some activities such as doing tasks, classroom participation, and interacting with teachers in terms of asking and answering questions are related to behavioral engagement. They also defined emotional engagement as learners' emotional reactions in classroom contexts. Moreover, they described cognitive engagement as learners' tendency to use complicated learning strategies instead of simple strategies. Reschly et al. (2020) asserted that behavioral engagement is significantly correlated with cognitive and affective engagement. The general agreement in the educational study is that the idea of engagement, as a whole, is critical but overlooked in the traditional school structure (Zhao Y. et al., 2021).

Learners' academic engagement includes individual and contextual features that interact with each other to demonstrate their positive attitudes toward learning (Zhao H. et al., 2021). Eccles (2016) argued that learner academic engagement has a positive and significant relationship with academic achievement and resilience. Evans and Tragant (2020) argued that learners with low levels of academic engagement can encounter critical problems like dropping out. Peng (2021) considered learner motivation a precondition for learner engagement and academic achievement. Lin (2022) also pointed out that learners' cultural and educational backgrounds and teacher attitudes toward the learner can affect their academic engagement and motivation. Ghelichli et al. (2020) found significant correlations between language learning motivation and each aspect of learner engagement. Their study also revealed the strongest significant correlation between cognitive engagement and language learning motivation.

Interactions with the teacher and their use of methodologies are also effective in academic engagement. Hashim et al. (2014) demonstrated that the interaction and rapport between teacher and learner influence learners' engagement. In another study, Sadoughi and Hejazi (2021) demonstrated that teacher support is directly related to academic engagement. Luan et al. (2020) investigated the prediction of online EFL learners' perceived social support in their engagement. They found out that the correlations between teacher support and learners' cognitive, emotional, and social engagements are mediated by learners' behavioral engagement. Guilloteaux (2016) asserted that learners' engagement is affected by their background level, task type, task difficulty, instructors' methodology, motivation, and their teaching style. Hung (2015) argued that memorization and rote learning, as two outdated teaching approaches, significantly predict learners' disengagement. He suggested flipped instruction for solving disengagement.

### The Role of Psychological Well-Being in Learners' Grit

Grit, as a non-cognitive skill, has drawn the attention of investigators. Non-cognitive behaviors can influence one's achievement or pleasure (Tough, 2012). Cross (2014) described grit as an ability to tolerate difficulties though preserving the wish for long-term purposes. According to Choi (2020), “grit, not only refers to resilience against failure, but also it covers an individual's tenacity in attaining an objective through incessant effort” (p. 144). Duckworth (2016) identified tenacity, growth, intrinsic motivation, and resilience as the components of grit. Thaler and Koval (2015) also argued that grit is inconsistent and can bring about required results via instructive and environmental interventions. Grit has a great influence on learners' academic achievements (McCain, 2017) and their language learning achievement (Robinson, 2015). In a study, Keegan (2017) revealed a significant correlation between grit and foreign language learning achievement. Based on her argument, “integrating more learner reflection with all [L2] classroom activities or assessments can help build grit” (p. 8). In the Chinese context, Wei et al. (2019) found out that grit has a significant role in improving EFL learners' reading, listening, and writing. Earlier studies demonstrated that grit is significantly correlated with learners' contentment (Von Culin et al., 2014) working memory (Eskreis-Winkler et al., 2014) willingness to communicate (Teimouri et al., 2020), test emotions (Datu and Fong, 2018), self-efficacy (Alhadabi and Karpinski, 2019), ideal L2 self (Feng and Papi, 2020), and self-confidence in language learning context (Lee and Hsieh, 2019).

Few studies have been done on the relationship between grit and subjective and psychological well-being in EFL contexts. Jin and Kim (2017) investigated the relationship between learners' grit, satisfaction with needs, and their subjective well-being. They found a negative correlation between grit and depression which may be the reason for the significant correlation between grit and subjective well-being. They also argued that learners' satisfaction with individuality requirements results in greater subjective well-being. Bono et al. (2020) investigated the role of grit in learners' apprehension and subjective well-being during COVID-19 pandemic. They found a significant relationship between grit and learners' subjective well-being. They argued that learners' resilience and grittiness can improve their subjective well-being and this can diminish their emotional damage. They also maintained that the improvement of grittiness among learners through increasing their persistence, flexibility, and steadiness can develop their psychological well-being. Nazari and Alizadeh Oghyanous (2021) investigated relationships among novice and experienced teachers' psychological well-being, job-related pressure, and grit in EFL contexts. Their study found out that correlations among EFL teachers' job-related pressure, well-being, and grittiness are higher and significant among novice teachers. Yang (2021) showed a significant correlation between EFL learners' enjoyment and grit. He mentioned that higher levels of grit are associated with higher levels of enjoyment. His study also revealed that grit is significantly correlated with well-being. Consequently, they argued that learners with high levels of grit are assumed to have high levels of capabilities as they focus on their affairs greatly and are not disappointed by the adverse situations. It can be concluded from his study that EFL gritty learners with high levels of engagement tend to have higher levels of well-being. Akbag and Ümmet (2017) also found a significant relationship between learners' subjective well-being with their grit. They attributed the learners' higher levels of psychological well-being to their independence, capability, and relatedness as three components of psychological needs. They also maintained that gritty learners who can achieve their objectives tend to have higher levels of subjective well-being. Datu et al. (2019) found out that learners with higher levels of grittiness are unlikely to suffer from depression. Therefore, gritty learners mainly give attention to their persistence and interest in order to attain their objectives and they concluded that achievement in reaching objectives is highly related to well-being. Tiittanen and Daukantaite (2014) believed that learners' grittiness can be influenced by their perceptions about the world's meaningfulness and these perceptions are related to their psychological well-being.

### The Role of Academic Engagement in Learners' Grit

Keegan (2017) pinpointed that effective instruction can be stimulated by grit in EFL/ESL contexts. Definitely, few investigations have been done on the effect of learners' academic engagement on their grit in language learning contexts (MacIntyre et al., 2019b). Robinson (2015) investigated the relationship between engagement and grit among nursing students. Her study revealed that course engagement is significantly correlated with learner's grit. Moreover, she found out that persistence of effort is significantly correlated with learners' behavioral engagement. Datu et al. (2016) also found out that perseverance in academic efforts has a negative correlation with learners' academic and behavioral disengagement. In a cross-sectional study, Hodge et al. (2018) investigated the mediated role of academic engagement in the relationship between learners' grit and academic achievement. They found a significant correlation between grit, academic achievement, and academic engagement. They argue that gritted learners have higher levels of engagement and this leads to achievement in academic contexts. Their results indicated that learners' grittiness is a pleasant feature and their academic engagement should be underscored in academic contexts in order to accelerate academic achievements. Suzuki et al. (2015) investigated the relationship between grit and work engagement among Australian learners. Their study revealed that grit is significantly correlated with work engagement and academic achievement. They justified their results by considering meticulousness and self-control which act as mediator factors. They mentioned that learners who have higher levels of both meticulousness and self-control are considered to have work engagement and grittiness. Dennison (2020) investigated learners' and teachers' perceptions about grit and engagement in math and comprehension. However, he found out that grit, academic engagement, and academic success are not strongly correlated with each other. Teachers perceived that they can moderately change learners' capability to learn math. On the other hand, learners had not strong self-confidence to use their grit in reading comprehension. Baquerizo (2018) did not find any significant relationship between grit and academic engagement. O'Neal et al. (2019) compared elementary learners' grit with their emotional engagement. They operationalized emotional engagement as learning enjoyment. They reasoned that motivation, as an important mediator, plays a key role in the significant relationship between grit and emotional engagement.

## Implications and Suggestions for Further Research

This review examined the related literature on the effect of learners' well-being and their engagement on their grit. It can be stated that learners through accentuating the related literature on the correlations among affective factors can be aware of controlling, adjusting, and regulating their feelings in language learning contexts. Learners can not only develop their success over the academic life, but also enhance their grit via developing their psychological well-being and academic engagement. Those learners who cannot regulate their feeling make teachers try to integrate psychological well-being into the EFL context through consulting with learners and providing pleasant learning contexts. They can also improve their grit by encouraging learners to engage in classrooms. L2 instructors should try to find materials to develop learners' positive attitudes and motivation for increasing their positive affectivity such as foreign language enjoyment, engagement, pride, etc., and to alleviate negative feelings such as communication apprehension and disengagement in their classes. Teachers need to reduce learners' communication apprehension, disengagement, and tension and they are required to improve grittiness regardless of instructive problems in language learning environments to enrich L2 learning experiences. This review suggests that teachers should attempt to develop interesting and enjoyable language learning tasks because interesting tasks can boost learners' cognitive resources and consolidate learning materials in their minds. Therefore, the provision of enjoyable tasks can contribute learners to productively control their learning. This can reduce learners' cognitive load and arouse their attentiveness in language learning contexts (Piniel and Albert, 2018). Furthermore, EFL teachers' knowledge about learners' characters may inspire instructors to be more stable and engaging in their behavior in language contexts.

As a teacher, we can develop learner grit by reading a book like “How Children Succeed: Grit, Curiosity, and the Hidden Power of Character” in the classroom which arouses learner grit. We can give our learners the grit scale questionnaire and allow them to fill it. Moreover, we can provide some video files like TED videos discussing the effect of decisions on grit. Through this, they are more engaged in educating themselves rather than waiting for educators to help them. Therefore, learner empowerment is a great strategy to develop grit. We can use examples in our lives by playing videos about studious men and women who are gritty and successful in their life despite their disabilities. Students are required to have viewpoints about complications to stop giving up, leaving, or failing in their life and losing hope. We can also develop learner mindset by talking about their dynamic mindset. We can discuss this issue in our classrooms by telling that learner with growth mindset is more successful than those who think that intelligence is fixed. Finally, teachers should live grittily to be a model for language learners. We should tell our perseverance against the problems and our own work ethic yells so loudly that learners know exactly what we think about grit. In order to improve learner well-being, we, as a teacher, should develop learner resilience in facing challenges for their future jobs. It is better to use innovative ways to improve learner well-being in educational contexts. The projects, lectures, conferences, and workshops put extra stress on learners. In order to lessen the pressure, introducing mindfulness lessons can be beneficial, and we can change our course assessment.

Teacher educators and mentors can practically consider the existent circumstances in language learning contexts and provide some techniques for teachers to improve learners' psychological well-being, grittiness, and academic engagement. They can hold workshops in pre-service and in-service teacher courses to talk about the importance of grit in language learning. Similarly, this review suggests that teacher educators should be careful about the vital role of learners' positive emotions such as psychological well-being and their engagement in language learning contexts. They should also provide some techniques to increase learners' enjoyment since the increase in learners' enjoyment leads to the reduction in learners' anxiety and development of language learning. It is recommended that educators be gregarious, humorous, and helpful to innovate inspiring language tasks that link students' language proficiency to their psychological well-being, grit, and their academic engagement (Dewaele et al., 2019). This review recommends that teacher educators should have a positive view toward teachers and learners and they should provide well-organized and inspiring teaching methodologies which can construct a positive context for language learning and increase learners' well-being, excitement, and enjoyment to engage in the classroom. Therefore, attending to learners' psychological well-being, motivation, academic engagement, and their grit may enhance students' feelings of approval which may develop collaboration among peers and foster teacher's rapport with students and ultimately arouse learners' sense of enjoyment. This review can also inspire school principals and educational policymakers to brood over EFL learners' traits and their negative and positive emotions. Policymakers can hold academic engagement workshops which assist instructors to know how to decrease learners' communication anxiety and intensify their psychological well-being, grittiness, and their academic engagement. They can create a positive learning context in which students can participate in positive behaviors. The significance of psychological well-being and academic engagement make consultants to broaden their programs to expand the effect of these positive variables on learners' grit. They can identify learners' sources of academic disengagement and the barriers of learners' well-being and grit. Material developers can also incorporate podcasts and videotapes into learning contexts to be applied by instructors to provide positive emotions among learners (Peng, 2019). Therefore, materials developers should arouse learners' grit and engagement through providing creative tasks.

This review has some suggestions for further research. Future studies can validate numerous measures of learners' psychological well-being, grittiness, and academic engagement. In order to follow up the effect of learners' psychological well-being and academic engagement on their grit, a longitudinal study can also be done. Furthermore, research needs to be done to study learners' academic engagement, psychological well-being, and their grit in various educational, local, national, and cultural backgrounds. Some investigations need to be done on learners' mindset and its relation with their grittiness, psychological well-being, and their academic engagement. Future studies should be conducted on the effect of different components of grittiness on learners' foreign language learning achievement, academic engagement, and communication apprehension. Other investigations can also be done on the relationship between different components of grit and classroom anxiety in foreign language learning. It can also be scrutinized with respect to teacher-student interpersonal variables in EFL/ESL contexts (Xie and Derakhshan, 2021).

Regarding psychological well-being, the relationship between EFL learners' well-being and their emotional intelligence experienced in EFL contexts can be examined in the future. Further research needs to be done on the effect of instructors' methodologies on learners' well-being. Furthermore, future studies can focus on gender's effect on language learners' well-being. Researchers pinpointed the effect of Digital Video Games on EFL learners' academic engagement (Boyle et al., 2012). Investigations are required to study the effect of video games on learners' well-being. Besides, future studies can inspect the effects of psychological well-being on the working memory of EFL learners. Also, the influences of EFL learners' well-being on the improvement of language skills should be cogitated in more detail. Moreover, the mutuality of the correlation between EFL learners' well-being and grit should be inspected in the future. The effect of learners' academic engagement on teachers' well-being can also be investigated. Online language learning has transformed language teaching approaches during the COVID-19 pandemic (Wang and Derakhshan, 2021). Future investigations should shed light on learners' psychological well-being in traditional and virtual contexts to elucidate the influence of learning contexts on the components of positive psychology. Multiple variables such as third language knowledge, study experiences abroad, and educational background and their relationship with learners' psychological and subjective well-being are required to be investigated.

Regarding academic engagement, further studies need to be done to clarify the relationship among foreign language learners' hope, optimism, engagement, and emotional intelligence. Furthermore, the relationship between learners' academic engagement and their self-efficacy and their effect of them on teacher burnout need to be investigated. Also, the relationships between learners' academic engagement and hope as positive psychology constructs and some negative emotional components such as anxiety and boredom are worth to be studied. Resilience, as another component of positive psychology, and its relationship with academic engagement should be inspected in more detail. Future research may consist of the relationship between learners' academic engagement and other individual variables such as EFL learners' extroversion and introversion.

## Data Availability Statement

The original contributions presented in the study are included in the article/supplementary material, further inquiries can be directed to the corresponding author.

## Ethics Statement

The studies involving human participants were reviewed and approved by Xi'an International University Academic Ethics Committee. The patients/participants provided their written informed consent to participate in this study.

## Author Contributions

The author confirms being the sole contributor of this work and has approved it for publication.

## Conflict of Interest

The author declares that the research was conducted in the absence of any commercial or financial relationships that could be construed as a potential conflict of interest.

## Publisher's Note

All claims expressed in this article are solely those of the authors and do not necessarily represent those of their affiliated organizations, or those of the publisher, the editors and the reviewers. Any product that may be evaluated in this article, or claim that may be made by its manufacturer, is not guaranteed or endorsed by the publisher.
